# Rain Area Detection in South-Western Kenya by Using Multispectral Satellite Data from Meteosat Second Generation

**DOI:** 10.3390/s21103547

**Published:** 2021-05-19

**Authors:** Kumah K. Kingsley, Ben H. P. Maathuis, Joost C. B. Hoedjes, Donald T. Rwasoka, Bas V. Retsios, Bob Z. Su

**Affiliations:** Faculty of Geo-Information Science and Earth Observation (ITC), University of Twente, 7500 AE Enschede, The Netherlands; b.h.p.maathuis@utwente.nl (B.H.P.M.); j.c.b.hoedjes@utwente.nl (J.C.B.H.); d.t.rwasoka@utwente.nl (D.T.R.); v.retsios@utwente.nl (B.V.R.); z.su@utwente.nl (B.Z.S.)

**Keywords:** rainfall detection, MSG SEVIRI, parametric model, cloud top properties, conceptual model, GPM IMERG

## Abstract

This study presents a rain area detection scheme that uses a gradient based adaptive technique for daytime and nighttime rain area detection and correction from reflectance and infrared (IR) brightness temperatures data of the Meteosat Second Generation (MSG) satellite. First, multiple parametric rain detection models developed from MSG’s reflectance and IR data were calibrated and validated with rainfall data from a dense network of rain gauge stations and investigated to determine the best model parameters. The models were based on a conceptual assumption that clouds characterised by the top properties, e.g., high optical thickness and effective radius, have high rain probabilities and intensities. Next, a gradient based adaptive correction technique that relies on rain area-specific parameters was developed to reduce the number and sizes of the detected rain areas. The daytime detection with optical (VIS0.6) and near IR (NIR1.6) reflectance data achieved the best detection skill. For nighttime, detection with thermal IR brightness temperature differences of IR3.9-IR10.8, IR3.9-WV73 and IR108-WV62 showed the best detection skill based on general categorical statistics. Compared to the Global Precipitation Measurement (GPM) Integrated Mult-isatellitE Retrievals for GPM (IMERG) and the gauge station data from the southwest of Kenya, the model showed good agreement in the spatial dynamics of the detected rain area and rain rate.

## 1. Introduction

Accurate rainfall detection and estimation in space and time are essential for resolving scientific questions, for operational purposes such as early warning, forecasting and the development of services and applications that affect and influence human welfare and agricultural production decisions at a farm-scale level [1,2,3,4]. Unfortunately, due to the high spatial and temporal variability of rainfall, accurate detection and estimation are still open research challenges.

Over the past decades, the use of geostationary weather satellites for rainfall detection and estimation has gained research attention because they can be used to retrieve area-wide distribution of this spatiotemporally varying phenomena at a high temporal resolution. Though the satellite based information for retrieving rainfall is restricted to the cloud top and has an indirect relationship with ground rainfall observations [5], some authors (e.g., [6]) have indicated that the information helps quantify rainfall because of the apparent relationship between rain duration and amounts.

In the past, most of the retrieval techniques (e.g., [7,8]) focused on the relationship between a single cloud top property (the cloud top brightness temperature (BT)) from infrared (IR) channel, rainfall probabilities and intensities—a technique that works best for intense cold convective clouds. However, the challenge lies in distinguishing non-raining cold cirrus clouds from the raining convective ones. The result is an overestimation of the detected rain areas and the corresponding rain rates [9]. While some of the retrieval techniques e.g., [8] consider screening out cirrus clouds, they usually require ancillary data (e.g., radar data), which is not always available everywhere. Furthermore, the single IR retrieval technique shows drawbacks when applied to detect and quantify rainfall from stratiform clouds. These clouds have spatially homogenous (warmer) temperatures that do not differ significantly between raining and non-raining clouds. Stratiform rains are usually of low intensities but with extensive coverage. As a result, a single IR based technique leads to uncertainties in the detected rain areas and rain rates [10]. IR-only satellite rainfall products such as [7] were developed for operational application in Africa; nonetheless, ground validation over the years have shown varying accuracies over the continent see, e.g., [11,12].

To overcome these downsides, several authors suggest the use of multispectral satellite data [9,13,14,15,16]. Multispectral satellites are passive remote sensing satellites that measure reflected energy within several specific regions (called spectral bands/channels) of the electromagnetic spectrum. Inoue and Aonashi [13] studied cloud information from visible and IR scanner and rain information from precipitating radar—both on-board the TRMM and GPM satellite for rain area detection. They showed that additional details from a second channel improve detection results compared to the single IR retrieval technique. In Thies, Nauss and Bendix [14,16], a method for discriminating from non-raining clouds during the day and night was proposed, combining visible and IR multispectral satellite data. Likewise, [9] demonstrated rain detection by utilising spectral and textural cloud features inferred from several IR channels. Finally, [15] showed, for the first time, rainfall detection in gauge scarce areas by using commercial microwave links (CML—the infrastructure used for telecom data transmission) and visible and IR multispectral geostationary satellite data.

Multispectral satellite based rainfall detection often applies parametric based techniques that relate cloud top information (e.g., cloud top temperature and height) derived from the satellite data to rainfall occurrences and rain rates see, e.g., [9,15]. The application is straight forward and requires a definition of the underlying conceptual models and parametric test. The advantage is that they directly map the conceptual knowledge of the rainfall process onto the retrieval using the satellite data as proxies [10]. In Feidas and Giannakos [9], Meteosat Second Generation (MSG) IR satellite data was investigated for rain area delineation using a parametric technique and a conceptual model in which cloud top properties such as optical thickness and phase were used to detect precipitating clouds. The daytime and nighttime precipitation process separation and rainfall intensity differentiation technique by [17] uses visible and IR data from MSG in a conceptual design in which convective clouds with higher rainfall intensities are characterised by their larger vertical extensions and cold top temperatures. However, the uncertainties in the detected rain areas are often significant, which calls for the need to develop new techniques for improving rain area detection.

The MSG satellite is a significant advancement over previous geostationary weather satellites. MSG is spin-stabilised; its wide range radiometer, Spinning Enhanced Visible Infrared Imager (SEVIRI), is a multispectral sensor, having eleven spectral channels of 3 × 3 km and one high resolution visible (HRV) channel of 1 × 1 km nadir resolution that observes the earth’s atmospheric state and dynamics every 15 min [18]. The measurement resolution of MSG allows for quasi-continuous observation of rainfall distribution in near-real-time. MSG’s high spectral resolution offers the opportunity to infer cloud top properties such as optical thickness, effective radius size, phase, pressure and height, which can be used for successful rainfall detection and estimation [9,15,19,20].

Therefore, motivated by the need for accurate rain area detection and the need to use multispectral data to improve rain area detection, this study utilised reflectance and IR data from the MSG SEVIRI satellite for rainfall detection with two primary objectives: (1) to investigate the satellite data’s suitability for detecting raining areas over topographically complex regions by evaluating multiple rainfall detection models, and (2) to develop a rain area correction technique for improving the detected rain areas. A rain detection model developed on the basis of a parametric threshold technique is the primary vehicle for this investigation. Compared to state of the art MSG-based parametric rainfall detection studies such as [9], which evaluated the satellite data’s ability to discriminate between raining and non-raining clouds, this study distinguishes itself on the following reasons: (1) we optimally searched for daytime and nighttime rain area detecting parameters from both optical and thermal IR data, and (2) we improved the detected rain areas using a newly developed gradient based adaptive technique. The structure of the paper is as follows. The data and method are presented in Section 2. The results are shown in Section 3 and discussed in Section 4. Finally, in Section 5, a conclusion on the significant findings is presented.

## 2. Data and Methods

### 2.1. Study Area

**Figure 1:**
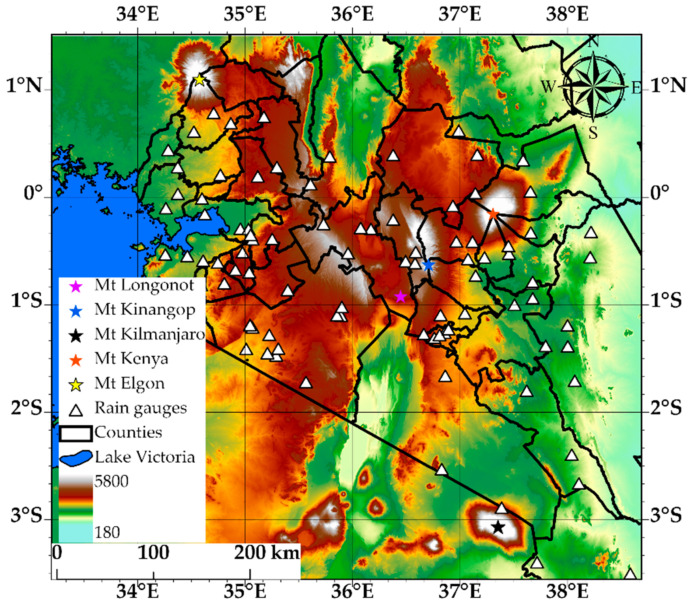
Study area and locations of rain gauges (triangles) displayed using ALOS DEM.

The area investigated (southwest Kenya) is shown in Figure 1 using Advanced Land Observing Satellite (ALOS) World 3D 30 m (AW3D30) digital elevation model (DEM) [19]. The main rainy season occurs from March to June (“long rains”), and the second season is from October to December (“short rains”). The rainfall variability in the area is generally linked to the seasonal passage of the intertropical convergence zone (ITCZ) over Kenya [20], tropical pacific sea surface temperature [21], El-Niño Southern Oscillation (ENSO) and the Indian Ocean Dipole (IOD) [22,23]. Nonetheless, relief features like mountains (Mount Kenya, Rift valley) and large inland water (Lake Victoria) bodies influence the local rainfall variability [24].

### 2.2. The Dataset

The dataset consists of MSG SEVIRI, GPM IMERG and rain gauge rainfall observations for the long rain period of 2018, 2019 and 2020. The MSG data was from the Meteosat satellite at longitude 41.5° E, including reflectance and IR channels sensitive to different cloud top properties, e.g., optical thickness, effective radius and phase during the day and night. These channels correspond to visible (VIS0.6 µm), near-infrared (NIR1.6 µm), thermal infrared (IR3.9 µm, IR8.7 µm, IR10.8 µm and IR12.0 µm) and water vapour (WV6.2 µm and WV7.3 µm) which were acquired at 15 min interval. The data are freely available at the European Organisation for the Exploitation of Meteorological Satellites (EUMETSAT) data archives [25]).

The rain gauge data was from the Trans-African Hydro-Meteorological Observatory (TAHMO). TAHMO maintains a network of ground-based weather stations across Sub-Saharan Africa, which can be accessed at [26]). These stations (see locations in Figure 1) measure standard meteorological variables such as rainfall, relative humidity, solar radiation and wind speed at 5 min intervals. [27]. The data has previously been used to ground validate the GPM IMERG satellite rainfall product over Africa [28]. This study used rainfall data from ninety stations (see their locations in Figure 1) that were distributed over the study area and had data during the evaluation period. TAHMO records rainfall accumulations every 5 min, which was used to estimate rain rates at 30 min intervals. It is also noteworthy that the TAHMO data are not part of the global network of rain gauges used by the Global Precipitation Climatology Centre (GPCC). Therefore, this study independently compared its results with the GPM IMERG final run version 6 (V06B) satellite global precipitation product, which is gauge calibrated with rainfall data from GPCC.

The IMERG final run version 6 (V06B) is the latest level 3 globally gridded satellite precipitation product derived from satellite radiometric observations from several GPM constellation satellites—consisting of a GPM Core Observatory satellite equipped with a dual-frequency precipitation radar and a 13-channel passive microwave (PMW) imager, and multiple partner satellites. The algorithm draws strength from previous satellite merging techniques such as the TRMM Multi-Satellite Precipitation Analysis (TMPA) [29]. In the algorithm, rainfall estimates from the constellation satellites and based on the Goddard Profiling Algorithm (GPROF2017) are first grided and intercalibrated to the estimates of the GPM Core satellite. They are then merged from their native spatial resolution on to IMERG grid at half-hourly times step. For areas with no PMW overpass and for beyond a forecast time of ±30 min from the closest PMW observation, IMERG uses the Climate Prediction Center (CPC) Morphing-Kalman Filter (CMORPH-KF) Lagrangian time interpolation scheme and the Precipitation Estimation from Remotely Sensed Information using Artificial Neural Networks Cloud Classification System (PERSIANN-CCS) re-calibration scheme to create the half-hourly estimates. Unlike previous versions, the motion vectors for the morphing are from hourly water vapour motion vectors from Modern-Era Retrospective Analysis for Research and Applications, version 2 [30,31]. Among the various IMERG precipitation products, this study utilised the MERG Calibrated precipitation estimates (precipitationCal), which is gauge calibrated with ground rainfall data from the Global Precipitation Climatology Centre (GPCC). The data is available freely at [32], with 10 km, 30 min resolution.

### 2.3. Method

#### 2.3.1. Spectral Characteristics and Cloud Top Properties

The method used for detecting raining clouds was based on investigating the relationship between the satellite data’s spectral characteristics, cloud top properties (Table 1) and rain. The conceptual idea used was that clouds characterised by their top properties, such as high optical thickness and effective radius (consisting of either ice or water hydrometeors), have high rainfall probability and intensities than those with low optical thickness and effective radius [14,15]. The physical basis of this assumption is derived from the following characteristics of raining clouds: (i) the availability of adequate moisture, (ii) an effective mechanism for converting small cloud droplets that are suspended in the atmosphere into raining particles and (iii) existence of ice phase clouds to support rain generation by the Bergeron–Findeisen process [33].

In this study, original reflectance, brightness temperature (BT) and brightness temperature differences (BTD) of the MSG satellite were used to infer cloud top properties [9,14,19] that detected raining areas under day and night conditions. Different combinations of the satellite channels were used for rain detection during the day and night periods because the MSG VIS0.6 and NIR1.6 µm channels are not available at night. Also, the use of IR3.9 µm during the day and twilight times is discouraged due to solar and thermal contributions and varying solar components in this channel [34,35]. The daytime and nighttime periods were from 04:15 AM to 3:15 PM and 4:15 PM to 03:15 AM UTC, respectively, excluding twilight periods between 03:30 AM and 04:00 AM and 3:30 PM and 4:00 PM UTC. The European Organization for the Exploitation of Meteorological Satellites (EUMETSAT) operational cloud mask product [36] was used to identify cloudy pixels to ensure that only satellite data from cloudy scenes were used in this study.

Several studies e.g., [15,16,19], have shown that the two reflectance channels: VIS0.6 and NIR1.6 µm, can be used to infer cloud top optical thickness and effective radius, respectively. Clouds with high optical thickness and effective radius (having either water/ice hydrometeors) have comparatively high VIS0.6 µm and low NIR1.6 µm reflectance than clouds with low optical thickness and effective radius. This is due to the non-absorption of the solar radiation at the VIS0.6 µm channel and the absorption of the radiation by the hydrometeors (higher in ice than water clouds) at the NIR1.6 µm channel [37,38]. Both properties (the cloud optical thickness and effective radius) point towards a single parameter, cloud water path (CWP), interpreted as the amount of water vertically integrated into the cloud and is directly linked to the rainfall probability of clouds. High VIS0.6 µm reflectance that coincides with low NIR1.6 µm reflectance implies clouds with high optical thickness and effective radius, and as such large CWP. In fact, the two reflectance channels have been successfully used for rainfall detection and estimation see e.g., [15,39]. Kuhnlein, Thies, Nauss and Bendix [39] studied the relationship between gauge rainfall and MSG’s VIS0.6 and NIR1.6 reflectance data using a conceptual model in which the two channels were considered in place of optical thickness and effective radius for rainfall-rate assignment. The results show encouraging performance, particular for temporal resolutions of 6 and 12 h, which may be considered better than those obtained based on a single IR channel. Based on a similar conceptual model, [15] investigated the combination of CML rainfall and the two reflectance channels for area-wide rainfall detection and rain rate retrieval. The results (over a reasonably small area) were convincing and revealed the potential of the two systems for area-wide rainfall monitoring to benefit gauge scarce areas or complement existing techniques.

The BT at IR10.8 µm channels is a good indicator of the cloud’s vertical extent because the BT of the cloud depends on the cloud top height [9,40]. Inoue and Aonashi [13] observed lower BT (less than 260 K) in 11 µm for raining areas identified by a precipitation radar.

The BTD between IR10.8 and WV6.2 µm have been previously used for cloud classification [41,42] and rainfall detection [43]. In the latter, clouds with BTD < 11 K for IR10.8–WV6.2 (an empirically determined threshold) were classified as raining.

The split window technique (i.e., BTD IR10.8–IR12.0) implemented by [44,45] can be used to gain information about cloud optical thickness, which is useful for discriminating optically thick cumulus clouds from optically thin cirrus clouds [9,44,45]. Optically thick cumulus clouds show small BTD because of their black-body characteristics. In contrast, optically thin cirrus clouds show a larger difference due to the differential absorption by ice crystals between the two channels [46].

The tripsectral BTD: IR8.7–IR10.8 and IR10.8–IR12.0 have been used to infer cloud phase information [15,16]. The absorption of solar radiation by cloud hydrometeors differs (for ice and water) between the two BTDs. Water particle absorption is stronger between 11 and 12 µm than between 8 and 11 µm, and for ice, the reverse is correct, based on the results by [47] using High-Resolution Infrared Sounder and Advanced Very High-Resolution Radiometer observations from NOAA-9 and a high spatial resolution MODIS Airborne Simulator (MAS) data. As a result, the difference between 11 and 12 µm (comparable to the IR10.8–IR12.0 BTD of MSG) of water clouds is higher than the coincident difference between 8 and 11 µm, whereas the difference between 8 and 11 µm (comparable to the IR8.7–IR10.8 BTD of MSG) of ice clouds is higher than that between 11 and 12 µm.

The emission of radiation at IR3.9 µm channel is dependent on the particle size, such that the large particles have high emission than smaller particles. This is because of the increased scattering that dominates the small particle regime, reducing their cloud emissivity. For the IR10.8 µm channel, this behaviour is less distinct. Therefore, the BTD for IR3.9–IR10.8 are higher for optically thick clouds with a high effective radius than for clouds with small particles [17,33].

The BTD for IR3.9–WV7.3 are similar to that of IR3.9–IR10.8, but with generally higher differences that can be explained by the diminishing effect of the water vapour absorption and emission in the mid-to-low tropospheric levels on the brightness temperature in WV7.3 µm channel [14,18].

Unlike WV6.2 µm, the WV7.3 µm channel is positioned at the edge of the water vapour absorption band (approximately 50 kPa). Thies, et al. [48] performed radiative transfer simulations for the spectral ranges of SEVIRI WV and IR channels from both cloud-free and variable cloud top heights. They indicated that for cloud tops below the tropopause temperature level, the WV6.2 µm BT are lower than the WV7.3 µm BT. In contrast, for cloud tops above the tropopause level, the WV6.2 µm BT are higher than that of the WV7.3 µm BT due to the stronger absorption lines of the WV6.2 µm channel. Based on these observations, it is anticipated that optically thick raining clouds with cloud tops piercing through the tropopause level will show small negative to positive WV6.2–WV7.3 BTD. For low-level clouds with cloud tops below the tropopause level, large negative differences may be observed.

#### 2.3.2. Data Pre-Processing

To develop the parametric rain detection models, rainy days were selected from the rain gauge datasets during the evaluation period. Rainfall over the study area is highly variable in space and time [24,49]; therefore, rainy days were identified separately per gauge station as a day with accumulated rainfall above 1 mm per day.

The MSG data over the study area did not require parallax correction because of the small zenith viewing angle of the Meteosat satellite (at 41.5° E) over the study area [15]. The satellite data were spatially aggregated. Both the satellite and rain gauge data were temporally aggregated to 30 min interval following the method described in [15] to reduce the effect of spatial and temporal mismatch between the satellite and gauge measurements in this study [39,50].

The resulting dataset consisted of collocated and coincident gauge rainfall and satellite reflectance and IR BT, which inferred cloud top properties for each rainy day at a gauge station. A cloud top property was then flagged as raining if the station rainfall was equal to or above 1 mmh^−1^, otherwise non-raining. The two sets of data, i.e., raining and non-raining cloud top properties, were later analysed separately to find the optimal parametric model parameter values discussed in Section 2.3.4.

Consequently, our dataset comprised gauge and satellite observations from a mixed space-time domain—implying that the dataset was derived from time-series observations sampled from different locations within the study area. As such, proper data splitting into calibration and validation sets [51] was needed to reduce spatial and temporal bias in the amount of data per gauge station used to train and validate the model and evaluate its performance on both seen and unseen rainfall events. Table 2 summarises the non-zero rain rates (i.e., above 1 mmh^−1^) from the calibration and validation sets.

#### 2.3.3. The Parametric Threshold Based Rainfall Detection Model

The rainfall detection method relies on a threshold applied to an m-dimensional space defined by the spectral characteristics that infer cloud top parameters. Here, we studied different combinations of cloud top parameters inferred from the reflectance, BT and BTD to determine the suitability of the satellite data for rain detection. It is worth mentioning that convective and stratiform rainfall are responsible for most of the area’s rainfall [52,53]. These two rainfall types differ in their spectral characteristics (particularly for the IR and BTD) used to infer the cloud properties and during the day and night conditions [13,17,38]. Nonetheless, we focused on the first primary objective and investigated different combinations of the BTD parameters (Table 3) for detecting rain areas suggesting that the developed approach does not consider the type of rainfall.

For a thorough evaluation, the rain detection model was categorised into 3 groups (Table 4), and the investigation was based on two major questions: (1) What is the rain detection skill of the satellite data if rain detection is based on reflectance-only, IR-only and combined reflectance-IR model? (2) Which model possesses the best rain detection skill? The model based on a single infrared (IR10.8) channel was included for comparison.

**Figure 2:**
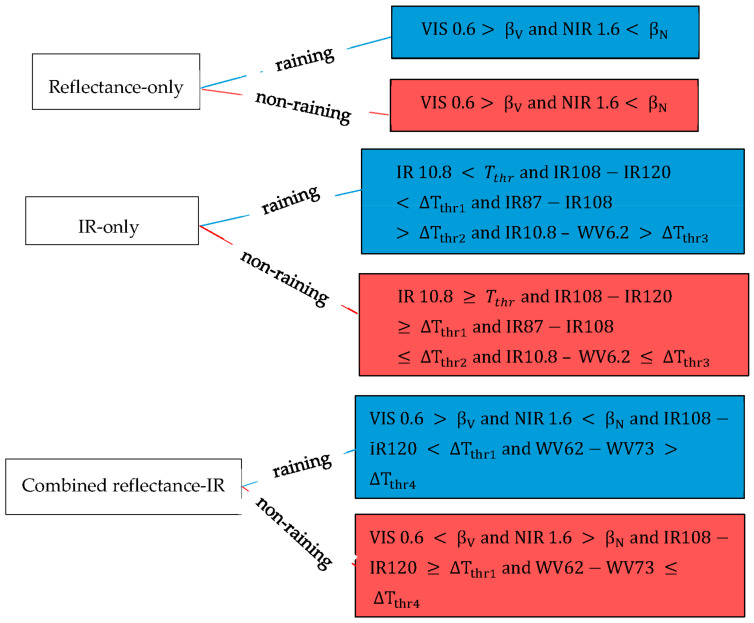
Examples of the dichotomous statement for each category of the rain detection model. The $\beta_{V}$ and $\beta_{V}$ are the thresholds for VIS0.6 and NIR1.6 reflectance, respectively, $T_{\mathrm{thr}}$ is the threshold for IR10.8 BT, $\Delta T_{\mathrm{thr}1}$, $\Delta T_{\mathrm{thr}2}$, $\Delta T_{\mathrm{thr}3}$, and $\Delta T_{\mathrm{thr}4}$ are the thresholds for IR10.8–IR12.0, IR8.7–IR10.8, IR10.8–WV6.2 and WV6.2–WV7.3 BTD, respectively.

For daytime, all 3 categories of models were evaluated, and for nighttime, the IR-only models were assessed due to the unavailability of the reflectance data. These models’ application assumes that a cloud is more likely to rain if the cloud top parameter is above or below a defined benchmark value. More precisely, in Figure 2, the model application is exemplified by dichotomous statements for raining and non-raining cases of, for instance, a reflectance-only, IR-only or combined reflectance-IR model.

A significant challenge to rainfall detection and estimation by the parametric threshold technique is non-raining thin/thick cirrus clouds because they result in erroneous estimates. The objective method for cloud-type classification (i.e., cirrus, thick cirrus, cumulonimbus and cumulus clouds) proposed by [45] was adopted to overcome this challenge. The basis for implementing this technique for cirrus clouds discrimination lies in the differential emissivity at 10.8 µm and IR 12.0 µm channels for cirrus clouds [54], leading to larger BTD for these clouds. Therefore in this study, the empirically derived threshold of 2.5 K for IR10.8-IR12.0 BTD [44,45] was used to screen out cirrus clouds before calibrating and validating our parametric rain detection models.

#### 2.3.4. Model’s Calibration and Validation

(I) Determining the best parametric model and parameters

The calibration of the various rain detection models (Table 4) was achieved by optimally searching for the threshold value of each model parameter in a range, e.g., x≤α≤y; where α is the parameter value and x, y are the upper and lower limits of the range of values, respectively. Each threshold value was used to conduct a raining/non-raining classification in the satellite data. The results were compared to the station data using categorical statistics to determine (1) the rain detection skill of the satellite based on the kind of satellite data used for rain detection and (2) the best rain detection model. A 2 × 2 contingency table (in Table 5) was used to define the frequencies of the model (satellite-based estimates) and gauge (reference/real observations) based on the raining/non-raining observations to compute some categorical statistics. The h in Table 5 are the raining observations detected in both the model and gauge observations (Hits). The m are raining observations detected by the gauge and not the model (Misses), and f indicate the those seen by the model and not by the gauge (False alarms). Finally, the frequency of non-raining observations detected by both the model and gauge observations was represented by z.

In Table 6, different categorical statistics were computed for each threshold value based on the contingency table elements. Each statistical parameter evaluates an aspect of the model performance. Collectively, they were used to find the threshold value that provides optimal model performance in rainfall detection. A detailed description of each statistical parameter can be found in, e.g., [55,56].

The different models were tested, and the best rain detection model was determined by optimising the value of the equitability threat score (ETS) together with the probability of detection (POD), false alarm ratio (FAR) and bias. The best model and corresponding threshold value appropriate for rain detection was defined as the model that maximises the ETS and POD values while minimising FAR and bias values.

(II) Validating the best parametric model and parameters

The best parametric models identified based on the daytime and nighttime statistical parameters were validated using the independent validation rainfall data. The validation approach was (1) by a point to pixel comparison of model rain detection with the gauge station data, (2) by comparing the model results to rain areas and rate from the GPM IMERG [57] satellite rainfall product and (3) by comparison of both model and IMERG detected rain rates to the gauge rain rates (i.e., the ground truth).

For the point to pixel validation, the best rain detection model and their corresponding parameters were used for rainfall detection, and the results were compared with the gauge rainfall data using all the statistical parameters in (Table 6). Note here that the rain area correction scheme described in the previous section was not implemented in this validation.

For the comparison with GPM IMERG, the model detected rain areas (that were corrected using the next section’s method) and rain rates were compared to the results from the latest IMERG Final Run version 6 (V06b) [58] because it is the latest improvement in the GPM IMERG satellite precipitation product. The focus was on the precipitationCal dataset because it is a research-grade product that is climatologically adjusted using ground data from the GPCC. Although recent studies see, e.g., [59,60,61] have ground validated and reported this new IMERG rainfall product’s performance elsewhere, its performance over gauge scare areas like the study area is generally not yet reported [62]. Therefore, this comparison intends to spatially validate the newly developed parametric model’s rain area and rate detection skill against the new IMERG V06b precipitationCal rainfall product.

The rain areas detected by the developed parametric model and IMERG were compared for the entire validation period. From IMERG, rain areas were identified by flagging IMERG pixels with a rain rate equal to or greater than 1 mmh^−1^ as raining, otherwise dry. IMERG has a 30 min and 10 km temporal and spatial resolution, respectively. To compare IMERG with the 3 km parametric model results from the MSG data, IMERG data was spatially resampled to 3 × 3 km using the nearest neighbourhood technique. The nearest neighbourhood resampling was used because it preserves the original pixel values as much as possible.

The rain rates detected by the developed parametric model and IMERG were compared at 30 min for the entire validation period. For the developed model, the detected rain rates were all the rain rates recorded at a gauge station when the model flagged the station pixel (i.e., the satellite pixel containing the gauge station) as raining. For IMERG, this corresponded to the rainfall retrieved from the IMERG pixel containing the gauge station. The absolute difference between the means of the two detected rain rates (i.e., the parametric model and IMERG) was compared per each gauge station and separately for the day and nighttime to assess the model’s daytime nighttime rain rate detection performances.

Finally, the probability density of the rain rates detected by the developed parametric model and IMERG, at 30 min for the entire validation period, were compared to the rain rate recorded at all the gauge stations. For this, the detected rain rates were organised into bins, and the number of rain rates in each bin was counted. The density was then computed as the count divided by the total count and the bin width. The cross-comparison purpose was to evaluate rain rate detection performance over the study area by the developed parametric model and IMERG against the ground truth and provide valuable insights that inform the ground validation wishlist provided by [62].

## 3. Results

### 3.1. Model Calibration

#### 3.1.1. Preliminary Analysis of the Spectral Characteristics of Cloud Top Properties

This section analyses the spectral characteristics of raining and non-raining clouds, which guided the determination of model parameter ranges described in Section 2.3.4. The analysis is presented separately for day and night times, and descriptive statistics of the data are in Appendix A (Table A1 and Table A2) and Appendix B (Table A3 and Table A4).

**Figure 3:**
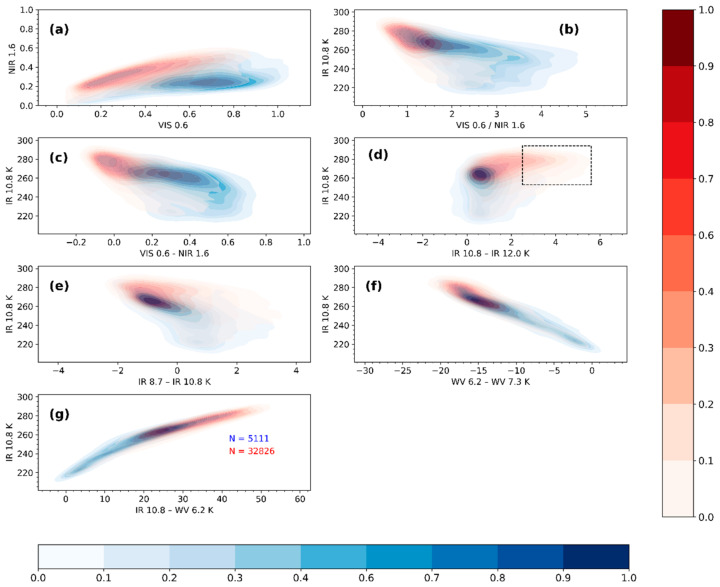
Bivariate probability density distribution of daytime raining (blue) and non-raining (red) spectral characteristics in (**a**) VIS0.6 and NIR1.6, (**b**) IR10.8 and VIS0.6 ÷ NIR1.6, (**c**) IR10.8 and VIS0.6–NIR1.6, (**d**) IR10.8 and IR10.8–IR12.0, (**e**) IR10.8 and IR8.7–IR10.8, (**f**) IR10.8 and WV6.2–WV7.3, (**g**) IR10.8 and WV6.2–IR10.8 space. The coloured figures in (**g**) are the raining and non-raining data counts, respectively, that computed the density distribution. The colour bar shows normalised densities to make the subfigures comparable with one another.

Figure 3 is a bivariate probability density distribution of the raining and non-raining spectral characteristics compared in a 2D-space for the daytime observations. Each figure’s contours represent the raining (blue contours) and non-raining (red contours) densities in the dichotomous dataset. A general observation from Figure 3 is the clear distinction in the peak of the distribution (i.e., the area in the plot where most of the data is concentrated; indicated by high densities) for the raining and non-raining densities. This characteristic behaviour is also supported by the significant difference in descriptive statistics (Appendix A, Table A1 and Table A2, respectively) computed from the raining and non-raining data. Although this observation is particularly noticeable in the reflectance than in the BT and BTD plots, it raises the possibility of rain and no-rain discrimination by using respective thresholds in the 2-D space.

In Figure 3a, one can notice that the raining cases of the spectral characteristics peak towards the lower right corner of the plot where large VIS0.6 (>0.6) reflectance coincide with low NIR1.6 (<0.4). Figure 3b,c also show higher (above 1.5 and 0.2 respectively) ratios and differences of the VIS0.6 and NIR1.6 reflectance together with colder IR10.8 BT (less than 265 K) for the raining cases. Also, the distribution tends to be bimodal with peaks above and below 250 K. The high VIS0.6 and low NIR1.6 reflectance and the corresponding high ratio and differences for the raining cases suggest that most of the raining cases defined by the rain gauges were from optically thick clouds with large CWP, high rainfall probabilities and intensities [15,39]. The first peak (IR10.8 > 250 K) in Figure 3b,c of the distribution for the raining cases suggest low level optically thick clouds, whereas the second peak (IR10.8 < 250 K) is indicative of high level optically thick cumulonimbus type clouds.

By contrast, the distribution of the non-raining cases shown in Figure 3a–c, all peak outside the area defined by the raining cases’ distribution. In Figure 3a, this corresponds to the lower left corner of the plot where low VIS0.6 (<0.6) reflectance coincide with low NIR1.6 (<0.4) reflectance. For Figure 3b,c, these correspond to areas in the plot where the reflectance ratio and differences are lower than their indicated respective thresholds and with IR10.8 BT that are mostly warmer than 265 K. From the figures, one can also notice that some non-raining cases overlap the areas defined by the raining cases. Based on the IR10.8 BT in Figure 3b,c, the areas defined by the non-raining cases mostly correspond to low and high level non-precipitating thin and thick cirrus clouds and N-Type clouds (representing edges of optically thick clouds, optically thinner cumulus clouds, or low-level cumulus cloud overlaid by thin cirrus clouds) according to the cloud type classification by [45,63].

Figure 3d similarly compares the distribution of the raining and non-raining cases for the IR10.8 BT and the split window BTD. The dotted square marks the IR10.8 BT and IR10.8–IR12.0 BTD threshold $\left(T_{\mathrm{thr}\;}>253\;K\;\mathrm{and}\;\Delta T_{\mathrm{thr}1}>2.5\;K\right)$ used to eliminate non-precipitating cirrus clouds (Section 2.3.3) according to the cloud classification technique by [44,45]. As can be seen from the plot, most of the raining cases are characterised by IR10.8–IR12.0 BTD < 1.5 K and IR10.8 BT colder than 265 K. Also, the distribution here is bimodal, having two peaks (above and below 250 K) with the IR10.8–IR12.0 BTD < 1.5 K. The first peak (IR10.8 > 250 K) comprises raining cases mostly from low-level cumulus clouds and the second peak (IR10.8 < 250 K) contains raining cases from mainly cumulonimbus type.

However, most non-raining cases are distributed above 1.5 K (IR10.8–IR12.0 BTD) and 265 K (IR10.8 BT). Note from the plot that some of the areas defined by the non-raining cases, especially above 265 K IR10.8 BT, overlaps with the raining cases. However, a large concentration of this overlap occurs in areas where the IR10.8–IR12.0 BTD > 1.5 K. Based on the cloud type classification presented by [63], most of these non-raining cases are from non-precipitating thin and thick cirrus clouds and N-Type clouds.

Figure 3e compares the raining and non-raining distributions of IR10.8 BT and IR8.7–IR10.8 BTD. Recall that the IR8.7–IR10.8 BTD has been used for separating water from ice clouds [47]. Larger IR8.7–IR10.8 BTD will mainly occur due to ice particles at the cloud top. It can be observed that most of the raining cases have IR8.7–IR10.8 K BTD above -2 K and colder than 265 K (IR10.8 BT). The distribution is again bimodal; first peaking above 250 K (IR10.8 BT) and IR8.7–IR10.8 BTD between −2 and 0 K and secondly, below 250 K and above 0 K for IR10.8 BT and IR8.7–IR10.8 BTD, respectively. The first peak consists of raining cases from mainly clouds with water droplets, while the second peak suggests clouds with ice tops–based on the threshold function presented by [64]. The non-raining cases are mostly warmer than 265 K IR10.8 BT and below 0 K for the IR8.7–IR10.8 K BTD. They are mostly cirrus (thin and thick) and N-Type clouds. It is also apparent from Figure 3e that some of the non-raining cases overlap with the areas defined by the raining cases.

The raining and non-raining distributions of IR10.8 BT and WV6.2–WV7.3 BTD are compared in Figure 3f. Most of the raining cases correspond to IR10.8 BT < 260 K and WV6.2–WV7.3 BTD > −18 K. The distribution also shows bimodal tendency, having two peaks above and below 250 K IR10.8 BT The first peak is between −18 and −10 K WV6.2–WV7.3 BTD and consist of raining cases from optically thick clouds below the tropopause level. The second peak (mostly having BTD above −5 K) comprises raining cases from optically thick cumulonimbus type clouds with cloud tops above the tropopause level. The non-raining cases, on the other hand, consist of N-Type clouds with WV6.2–WV7.3 BTD less than −15 K and IR10.8 BT warmer than 260 K.

Figure 3g presents a comparison of the raining and non-raining distributions for IR10.8 BT and IR10.8–WV6.2 K BTD. The raining cases consist of IR10.8 BT colder than 265 K and IR10.8–WV6.2 BTD mostly less than 30 K. From Figure 3g, the empirical threshold (<11 K) determined by [43] is consistent with raining cases from clouds with cold tops (<250 K). However, raining cases from clouds with relatively warm top temperatures (<265 K) show BTD mostly above the empirical threshold. On the other hand, the non-raining cases mainly consist of clouds with warmer top temperatures (IR10.8 BT > 265 K) and IR10.8–WV6.2 BTD > 30 K.

**Figure 4:**
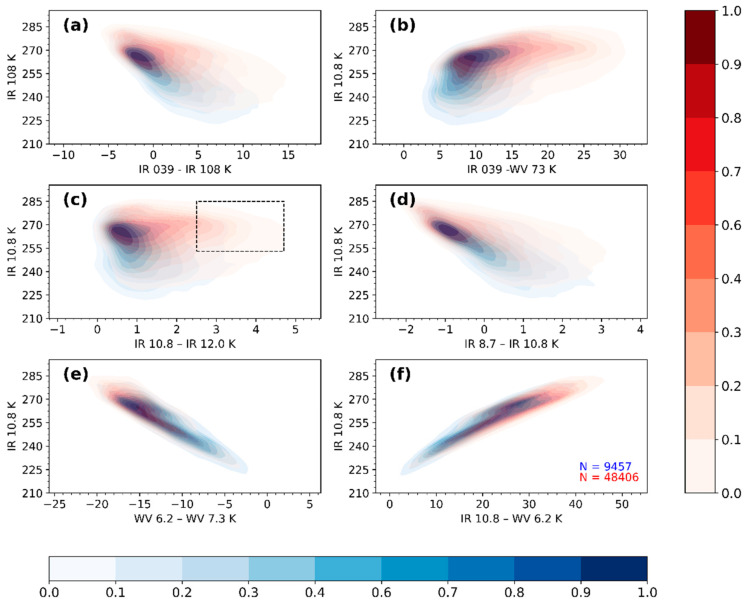
Bivariate probability density distribution of nighttime raining (blue) and non-raining (red) spectral characteristics in (**a**) IR10.8 and IR3.9–IR10.8, (**b**) IR10.8 and IR3.9–WV7.3, (**c**) IR10.8 and IR10.8–IR12.0, (**d**) IR10.8 and IR8.7–IR10.8, (**e**) IR10.8 and WV6.2–WV7.3, (**f**) IR10.8 and WV6.2–IR10.8 space. The coloured figures in (**f**) are the raining and non-raining data counts, respectively, that computed the density distribution. The colour bar shows normalised densities to make the subfigures comparable with one another.

Figure 4 is analogous to Figure 3 but for nighttime. Here, unlike Figure 3 (the daytime data), one can observe a significant overlap in the raining and non-raining distributions. Their descriptive statistics (Appendix B, Table A3 and Table A4) also paints a similar picture based on the comparatively similar statistical parameter values. Additionally, the figure shows bimodally distributed densities of IR10.8 BT above and below 255 K, which is slightly warmer than temperatures observed in the daytime data.

Figure 4a shows a comparison of the raining and non-raining distributions for IR10.8 BT and IR3.9–IR10.8 BTD. The raining cases mainly consist of IR3.9–IR10.8 BTD between 0 and 5 K, and IR10.8 BT colder than 270 K. These spectral characteristics are consistent with medium BTD found in [14,15] and are indicative of large CWP with high rain probabilities and intensities. The non-raining cases show mostly large positive differences in clouds with medium CWP and low rain probability and intensity.

The results for comparing the raining and non-raining IR10.8 BT and IR3.9–WV7.3 BTD (Figure 4b) show comparable characteristics to Figure 4a but with generally higher IR3.9–WV7.3 BTD. The higher differences are due to the diminishing effect of the water vapour absorption and emission in the mid-to-low tropospheric levels on the brightness temperature in the WV7.3 µm channel.

Figure 4c compares the raining and non-raining distributions for the IR10.8 BT and the split window BTD. Like in Figure 3d, the dotted square indicates the IR10.8 BT and IR10.8–IR12.0 BTD threshold used to eliminate non-precipitating cirrus clouds (Section 2.3.3). The IR10.8–IR12.0 BTD defined by the raining cases is mostly between 0 and 2 K and colder than 270 K IR10.8 BT. As noted earlier, these characteristics indicate optically thick cumulonimbus clouds. On the other hand, the non-raining cases are from non-precipitating cirrus clouds and N-Type clouds. Unlike the daytime observations (Figure 3d), the IR10.8 BT and the IR10.8–IR12.0 BTD are distributed over a wide range.

For the comparison of IR10.8 BT and IR8.7–IR10.8 BTD (Figure 4d), the observations made for the raining cases were comparable to those found during the daytime (Figure 2e). However, the non-raining cases showed a relatively wide range of IR10.8 BT values that mainly were warmer (>270 K).

Figure 4e,f compare the raining and non-raining distributions for IR10.8 BT and WV6.2–WV7.3 BTD and IR10.8 BT and IR10.8–WV6.2 BTD, respectively. Here, the observations made for both the raining and non-raining cases were comparable to their daytime observations (Figure 3f,g respectively), except their IR10.8 BT tend to show a wide range of values.

#### 3.1.2. Determination of the Optimum Parametric Thresholds

**Figure 5:**
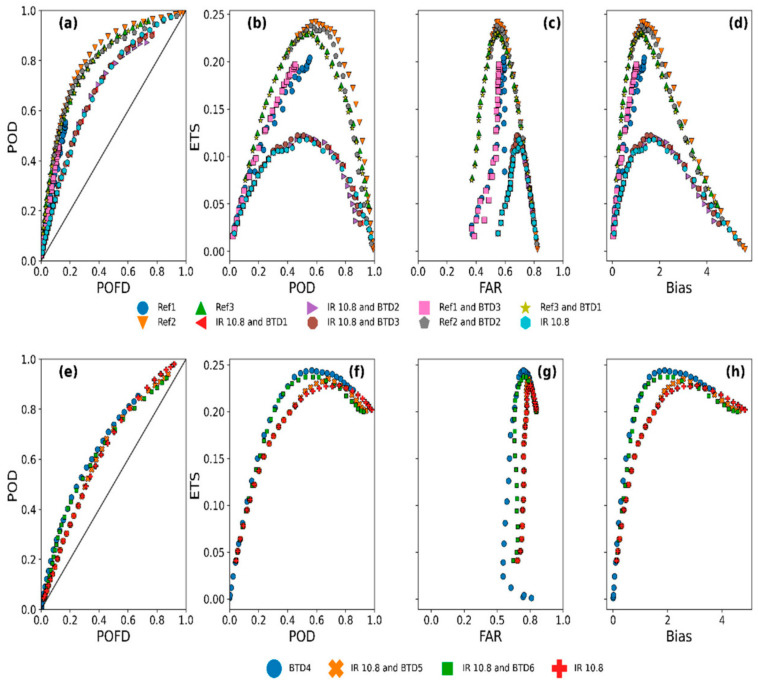
Daytime (**a**–**d**) and night (**e**–**h**) model calibration results. (**a**,**e**) ROC curve for different model parameter threshold. Statistical scores of ETS compared to (**b**,**f**) POD (**c**,**g**) FAR and (**d**,**h**) Bias.

Figure 5 presents the graphical representation to determine the parametric thresholds with the best rain detection performance to answer the 2 essential questions: the satellite data’s rain detection skill based on the kind of data and the best rain detection model. The categorical statistics of POD, POFD, FAR and bias were computed as a function of parameter thresholds to assess model performance and answer these two question for both the day and nighttime analysis. The POD versus the POFD was analysed to infer the models’ relative operational characteristic (ROC) curve—a measure of a forecasting system’s relative skill and usefulness [65]. The distance of the ROC curve from the diagonal line (where POD = POFD) corresponds to a climatological skill and is often used to evaluate the quality of forecasts [9].

From the daytime results (Figure 5a–d), the ROC curves (Figure 5a) for the different parametric thresholds of the rain detection models show that threshold values corresponding to models derived from the reflectance data have the largest distance from the diagonal line. Notably, the models derived from the VIS 0.6 and NIR 1.6 reflectance (Ref 2 and Ref 3) showed the best performance. This suggests that daytime rain detection based on reflectance measurements alone may be enough to achieve maximum detection results. The rain detection models developed from a combination of reflectance and IR showed medium performances, whereas those from the IR data alone were often poor.

The observations above are further supported by the relationship between ETS and the POD, FAR and bias values (Figure 5b–d respectively). From Figure 5b, it can be seen that the Ref 2 and Ref 3 models have comparatively higher ETS and POD values. Nonetheless, all models have reasonably high FAR and Bias (Figure 5c,d, respectively). It can also be seen from the figure that the rain detection models developed from combined reflectance and IR data tend to reduce model FAR and bias values but at a cost to the POD values.

To answer the question “which is the best parametric rain detection model?” the values of all 4 categorical scores were considered. Between the Ref 2 and Ref 3 models, the Ref 3 model was considered the best model because of its high ETS and POD and correspondingly low FAR and Bias values.

The nighttime results (Figure 5e–h), the ROC curves (Figure 5e) show that threshold values corresponding to BTD4 have the largest distance from the diagonal line, suggesting the best model performance. On the other hand, rain detection models developed from IR (IR10.8) BT and BTD usually resulted in medium model performance. In contrast, those based on a single IR BT often showed poor performance.

Moreover, when the ETS is compared to the POD, FAR, and Bias, the BTD4 rain detection model’s superior performance is further strengthened. Its ETS and POD values are higher than those for a combined IR and BTD and single IR models, with the single IR model being the lowest. Again, all models show higher FAR and Bias, although the Bias are comparatively lower than those found during daytime. Therefore, the BTD4 rain detection model is considered the best model for the nighttime case because of its high ETS and POD and comparatively low FAR and Bias values.

**Figure 6:**
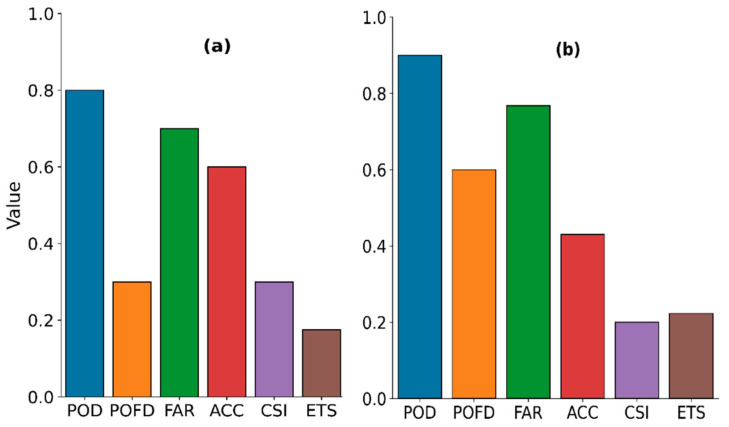
Categorical statistics from daytime (**a**) and nighttime (**b**) calibration of the best parametric rain detection model with gauge rainfall data.

The parameters and values for the best model are shown in Table 7, and their categorical statistics from the model calibration are presented in Figure 6. The daytime parameter value is comparable to the one inferred from the raining spectral characteristics (i.e., the satellite signals sampled when rainfall in the gauge was equal to or above 1 mmh^−1^) in the bivariate distribution, Section 3.1.1 (Figure 2c). In contrast, the nighttime parameters’ values are comparable to the sum of their 75th percentile and standard deviation values (Appendix B Table A3) of the raining spectral characteristics’ descriptive statistics.

Figure 6 shows a clear difference in rain detection performance between the daytime (Figure 6a) and nighttime (Figure 6b). Although their POD, FAR, and ETS are comparable, the comparatively high ACC, CSI, and low POFD and bias (2.28 compared to 3.67 for the nighttime case) scores suggest that the daytime detection was better than the nighttime.

### 3.2. Parametric Model Validation

This section validates the developed rain area detection and correction technique using the independent validation datasets for both the daytime and nighttime and the entire evaluation period. First, the point to pixel validation of the model results using the gauge station data is presented. Next, the developed rain area correction technique is described and demonstrated for two selected daytime (on 14 April 2018 11:00 UTC) and nighttime (on 6th March 2018 17:00 UTC) periods from the validation dataset due to the variety of detected rain areas present in the scene. Finally, both the model detected rain areas and rain rate are compared with the GPM IMERG satellite rainfall product results to validate the model spatially.

(I) Point validation of the parametric model’s rain area

**Figure 7:**
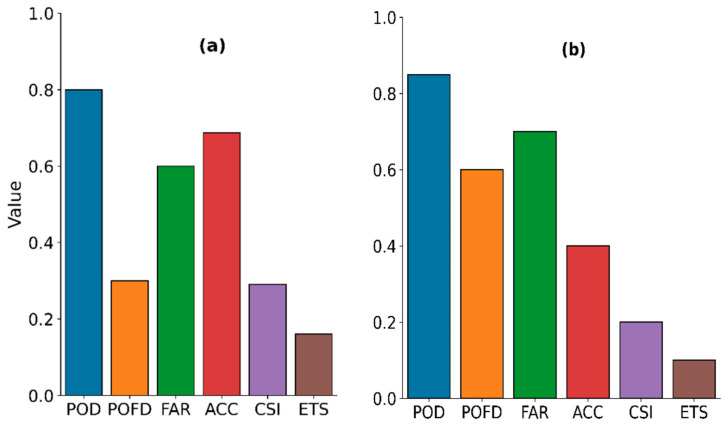
Categorical statistics from daytime (**a**) and nighttime (**b**) validation of the best parametric rain detection model with gauge rainfall data.

Figure 7 presents the categorical scores of rain area detection, indicating the best rain detection models’ performance when the model results were compared with the daytime (Figure 7a) and nighttime (Figure 7b) gauge station data. The results show improved daytime detection, indicated by high ACC, low FAR and a marginal increase in bias (2.3) scores compared to the calibration scores. However, the low ETS and high bias (4.11) scores, compared to its previous calibration scores, suggest a decreased model performance for the nighttime.

(II) The rain area correction scheme

Corrections were applied to the detected rain areas because the indirect relationship between rain and the inferred cloud top properties from the satellite often results in retrieval uncertainties [66]. Moreover, both the point validation of the initial rain area detection results (shown above) and its preliminary comparison with the results from EUMETSAT’s Multisensor Precipitation Estimate (MPE) [67,68] (not used in this study) and GPM IMERG (used in this study) showed high FAR and comparatively extensive raining areas, respectively, which required corrections. Below is a summary and results that detail the implementation of the correction technique.

This study implemented rain area correction for only the validation data because of the large number of datasets. The correction scheme relies on adaptive parametric thresholds applied to spectral characteristics from the detected rain area. This implies that for each classification scene and detected rain area, the applied corrections were based on scene and rain area-specific parameters. They were more precisely derived from the gradient in the spectral characteristics computed for each rain area, i.e., the identified raining cloud object (cloud object gradient) and each pixel (pixel gradient) in a cloud object. These two kinds of gradients differ for daytime and nighttimes due to the different data and information content used for the rain area detection. Nonetheless, their implementations are for the same purpose during day and night.

Cloud objects gradient aim to reduce the number of detected rain areas by combining the gradient computed for each cloud object (i.e., the detected raining area) with the average gradient and standard deviation from all cloud objects to locate non-raining areas previously classified as raining in the initial results. The number of detected rain areas herein refers to the number of areas (of different sizes) identified by the parametric model as raining. The pixel gradient is resulting in a reduction of the size of the cloud object because it compares the gradient computed for each pixel in a cloud object to its median and average median (from all cloud objects) to locate the non-raining high/low gradient (depending on the day/night application) pixels in the initial results.

**Figure 8:**
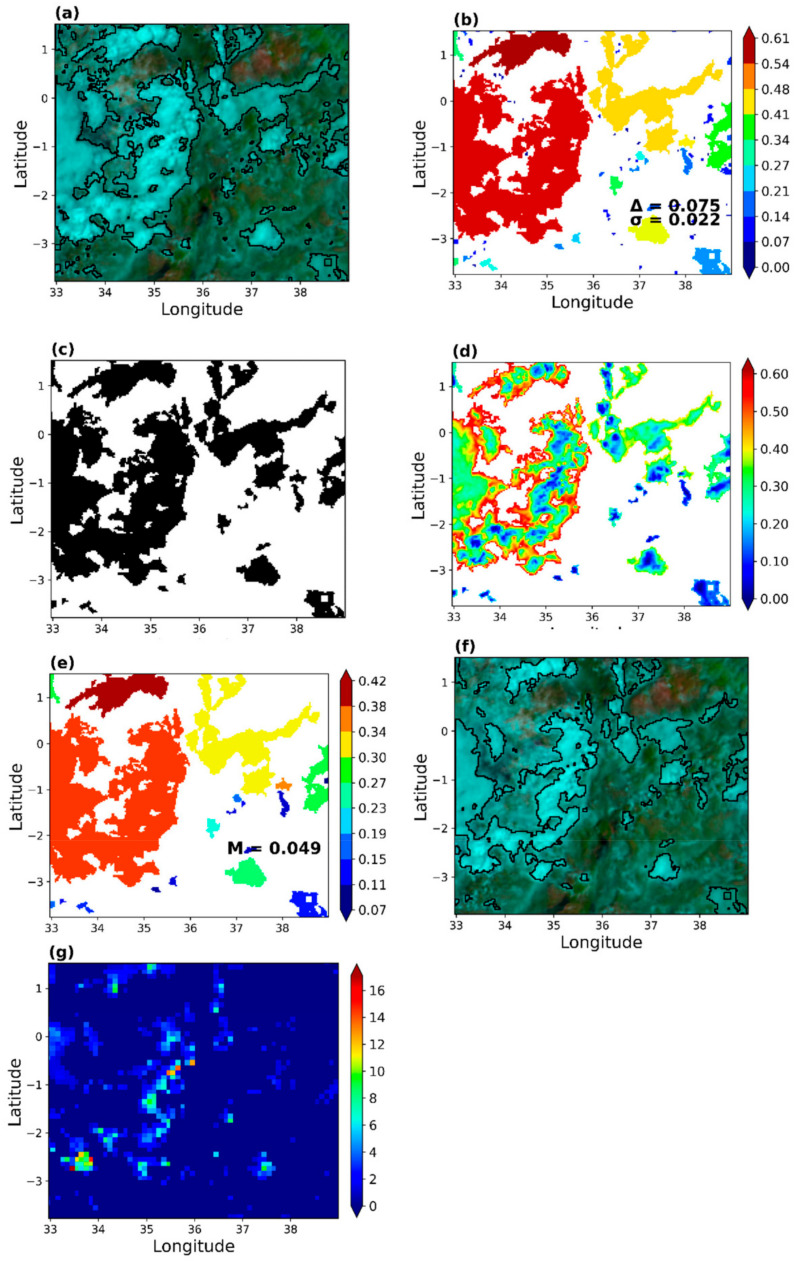
Daytime rain area correction. (**a**) Initial rain area detections, (**b**) Cloud object gradient, (**c**) Rain area correction based on cloud object gradient, (**d**) Pixel gradient, (**e**) Median pixel gradient, (**f**) Rain area correction based on pixel gradient, (**g**) GPM IMERG rainfall estimate (mm/30 min) over the study area.

Figure 8 demonstrates the implementation of the daytime rain area correction scheme. The best daytime model was determined to be the VIS0.6 − NIR1.6 parametric model (Ref 3). As was shown in Section 3.1.1 (Figure 3c), the raining spectral characteristics (i.e., those sampled when rainfall in the gauge was equal to or above 1 mmh^−1^) were mainly above 0.2, suggesting that higher differences correspond to high rain probabilities.

Figure 8a is a Daytime Natural Colour RGB [69] composite of SEVIRI NIR1.6, VIS0.8 and VIS0.6 µm channels, respectively, over the study area. High reaching clouds with ice tops, e.g., cumulonimbus type clouds, appear cyan in the figure. Black lines demarcate the raining areas initially detected by the developed daytime parametric model. The cloud object gradient (Figure 8b) represents an area-specific gradient computed (for each detected raining area in Figure 8a) as the maximum reflectance difference (max(VIS0.6–NIR1.6)) of each cloud objects minus its minimum (min(VIS0.6–NIR1.6)). The cloud object gradient is combined with the average gradient (Δ) and standard deviation (σ), both indicated in Figure 8b, to identify non-raining areas in the initial results and thus reduce the number of detected ted cloud objects (Figure 8c).

The pixel gradient (Figure 8d) was computed for each cloud object in Figure 8c as max (VIS0.6-NIR1.6) minus the pixel value. Thus, the low gradients in Figure 8d correspond with high VIS0.6–NIR1.6 differences, whereas the high gradients are the low differences. These gradients, combined with the median pixel gradient (Figure 8e) for each cloud object and the average of the median (M) pixel gradient from all cloud objects, identify and reclassify high gradient pixels as non-raining. The result is a reduction in the detected rain area’s size, as shown in the RGB colour composite in Figure 8f. Figure 8g is the GPM IMERG rainfall estimate over the study area. Comparing the initial results in Figure 8a–f (the corrected version) and the rainfall estimates in 8g, it is evident that the correction scheme can improve the initial results to estimates that are comparable with the GPM IMERG satellite estimates.

**Figure 9:**
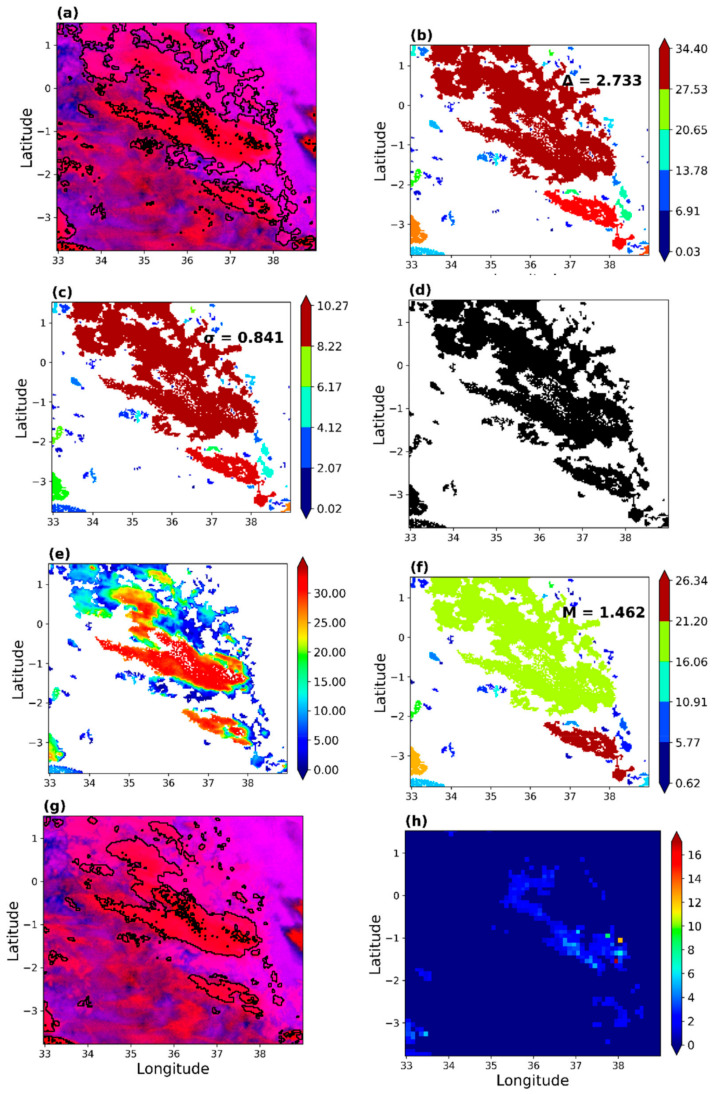
Nighttime rain area correction. (**a**) Initial rain area detections, (**b**) Cloud object gradient, (**c**) Cloud object standard deviation (**d**) Rain area correction based on cloud object gradient, (**e**) Pixel gradient, (**f**) Median pixel gradient, (**g**) Rain area correction based on pixel gradient, (**h**) GPM IMERG rainfall estimate (mm/30 min) over the study area.

Figure 9 demonstrated rain area correction for the nighttime. Unlike the daytime, nighttime rain detection was based on a combination of IR3.9–IR10.8, IR3.9–WV7.3 and IR10.8–WV6.2 BTD (BTD4). Here, the rain area-specific parameters used in correcting the detected rain areas were from IR10.8–WV6.2 K to reduce redundancy in the data used for the correction. Figure 3g and Figure 4f indicate that most of the raining spectral characteristics of the IR10.8–WV6.2 are less than 30 K (in Table A1 and Table A3, on average, 75% are <26 K), suggesting that low differences indicate high rain probabilities.

Figure 9a is a Nighttime Microphysics RGB colour composite of SEVIRI IR12.0 –IR10.8 µm and IR10.8–IR3.9 µm channel differences, and IR10.8 µm channel, respectively, over the study area. Detailed colour interpretation is provided in [69]; of interest is the reddish-brown areas that indicate optically thick ice clouds. The initially detected rain areas by the nighttime parametric model are demarcated in black. The cloud object gradient (Figure 9b) and Δ were computed similarly to the daytime. The cloud object standard deviation in Figure 9c and the σ (shown in Figure 9c) represents an area-specific standard deviation and σ of the IR10.8–WV6.2 BTD for the detected raining area. Combined with Figure 9b, they were used to reduce the number of detected cloud objects as shown in Figure 9d, similar to the daytime approach. The pixel gradient in the IR10.8–WV6.2 BTD for the areas in Figure 9d is also shown in Figure 9e. It was computed similar to the daytime approach. Thus, the high gradient area corresponds to the low IR10.8–WV6.2 BTD and vice versa. The median pixel gradient and the M shown in Figure 9f were derived from the raining areas in Figure 9e. They were used to reduce the sizes of the detected rain areas, as shown in Figure 9g. Figure 9h is the rainfall estimate from the GPM IMERG; its comparison with the initial and final (the corrected version) rain areas result (Figure 9a,g, respectively) provided a better perspective of the effect of the developed correction scheme similar to the daytime.

(III) Spatial validation of the parametric model’s rain area and rate

**Figure 10:**
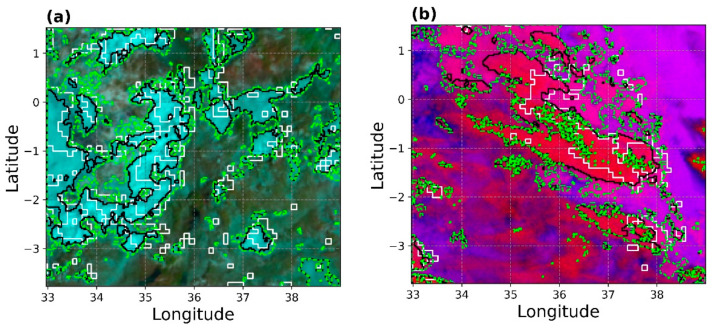
Spatial verification of the corrected rain area detections (black extent) compared to the initial detections (lime green) and GPM IMERG (white extent) for the day (**a**, on 14 April 2018 11:00 UTC) and nighttime (**b**, on 6th March 2018 17:00 UTC). The base maps are composite for Daytime Natural Colour (**a**) and Nighttime Microphysics (**b**).

Figure 10 is an “eyeball” verification of the corrected rain areas (Figure 8f and Figure 9g) compared with the initial rain areas detections (Figure 8a and Figure 9a, lime green demarcations) and detections by the GPM IMERG (white demarcations) for the daytime and nighttime in an RGB colour composite to validate the developed parametric model spatially. The daytime comparison in Figure 10a shows a convincing agreement in the detected rain areas’ spatial dynamics by the model and IMERG. For instance, areas between latitudes 0 to −2 and longitude 34 to 36 show a good spatial match in rain areas. These observations correlate with the high POD, ACC and CSI scores observed for the daytime in the previous validation, thus indicating high confidence in the model results.

Nevertheless, there are some differences in Figure 10a. IMERG detects more rainy areas (of varying sizes) than the developed model. Instead, the raining rain areas detected by the model are mainly organised into large areas and fewer in number. Additionally, a close inspection of Figure 10a reveals a slight shift in the detected rain areas by IMERG relative to the model.

Figure 10b is an analogous comparison of Figure 10a but for the nighttime. Compared to IMERG, the corrected rain areas’ spatial dynamics shows good agreement, especially for the large rain areas. However, the figure also shows that the nighttime detection shows some tendency to detecting more rainy areas than IMERG, which may explain the high POFD, low ACC and high bias scores observed in the previous validation. Moreover, the spatial shift in the detected rain areas of IMERG relative to the parametric model observed for the daytime is again noticeable.

The differences in rain areas detected by IMERG and the parametric model were mainly attributed to factors such as differences in the native resolution of the model dataset and IMERG. The spatial and temporal aggregation method used to match the two datasets could also further contribute to these differences.

Table 8 presents descriptive statistics of the detected rain areas’ spatial properties; herein, the number and size (i.e., the area in km^2^) for the uncorrected parametric model, corrected parametric model, and IMERG. The number of detected rain areas expresses the average count of all detected rain clouds, the 50% percentile, 75% percentile and standard deviation per 30 min validation timestep. On the other hand, the area in Table 8 expresses the average size, 50% percentile, 75% percentile and standard deviation of the clouds detected per 30 min validation timestep. Based on the Table 8 results, IMERG, on average, detects a comparatively smaller number of rain clouds of large sizes than the parametric model, which may be because of IMERG’s larger native spatial resolution than the model.

**Figure 11:**
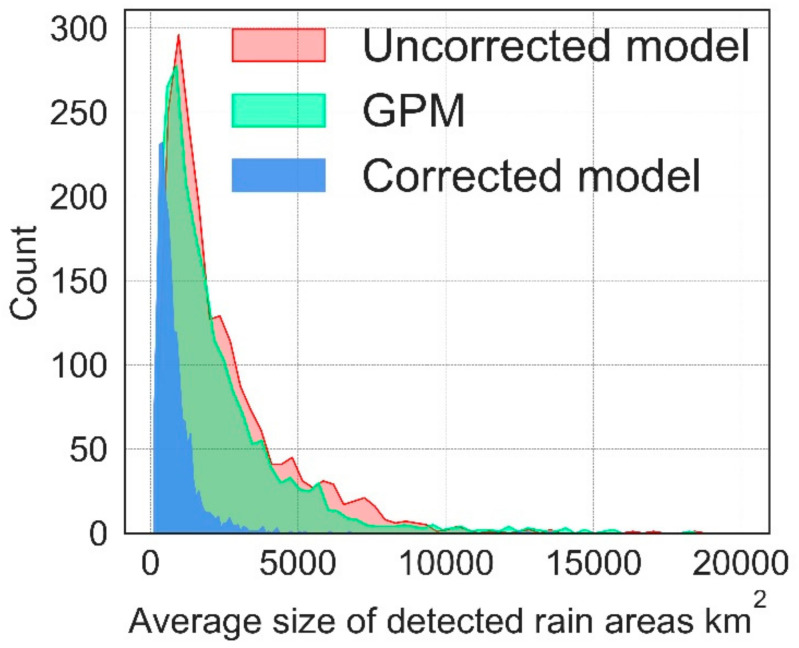
Comparing the average sizes of detected rain rates by the parametric model and IMERG.

Figure 11 compares the average sizes of the parametric model’s detected rain areas before and after correction to rain areas from IMERG for the entire validation period. The figure shows the average detected rain area for sizes ranging from 81 to 20,000 km^2^ because it constitutes most of the detected rain area sizes, and it allows for a clear visual comparison.

In general, the initial model results detected extensive rain areas of varying sizes. Nonetheless, the implemented correction technique was effective in reducing the number and sizes of the detected rain areas. From visual inspection of uncorrected and corrected detected rain areas, the daytime rain area correction mainly occurred at the fringes of initially detected large contiguous raining areas, whereas the small areas were mainly reclassified non-raining. On the contrary, the nighttime implementation showed that large areas initially flagged as raining were reclassified as non-raining.

**Figure 12:**
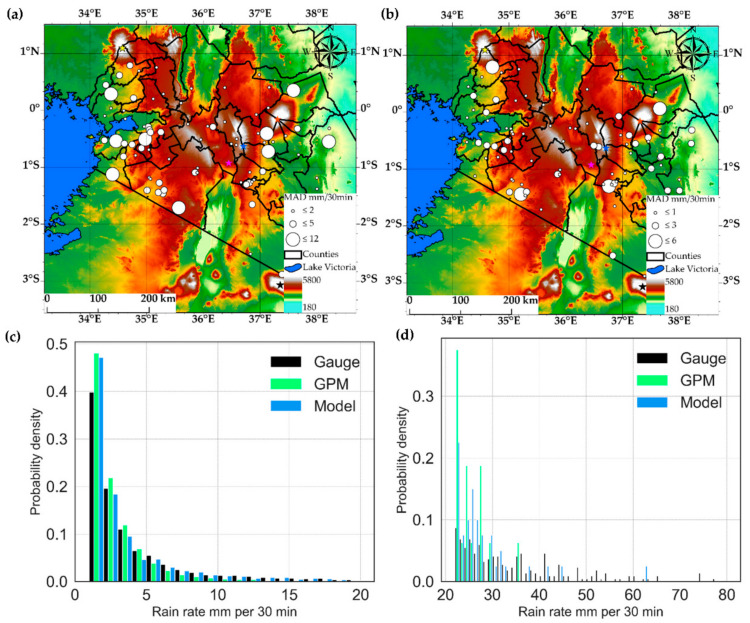
Comparison of the detected rain rates by the gauge, parametric model and IMERG. (**a**) Daytime absolute differences between the means of the parametric model and IMERG, (**b**) Nighttime absolute differences between the means of the parametric model and IMERG, (**c**) Probability density of below 20 mm per 30 min rain rates, and (**d**) Probability density of above 20 mm per 30 min rain rates detected by the gauge, IMERG and model.

Figure 12 compares the gauge stations’ detected rain rates to those detected by the parametric model and IMERG for the entire validation period, using absolute differences and probability densities. Figure 12a,b are the absolute differences between the mean rain rates detected at each gauge station by the parametric model and IMERG compared separately for the daytime and nighttime, respectively. The probability densities of the rain rates from the gauge, IMERG and parametric model were also compared for rain rates below and above 20 mmh^−1^ (Figure 11c,d) for the entire validation period to evaluate the models’ detected rain rates against the actual observations) and IMERG.

Figure 12a shows spatially varying daytime mean differences especially around mountainous areas (e.g., between Mount Kenya and Kinangop) and Lake Victoria, where the highest differences (<12 mmh^−1^ on average) were observed. These areas coincide with areas previously identified with high spatially varying rain rates [49,70] and differences between satellite and gauge rain rates [1,71] which may be due to the extreme precipitation observed in these areas [72] and the influence of orography and large water body on rainfall variability [24,71,73]. In particular, mountains pose a unique challenge to PMW rainfall retrievals because their rainfall signal over land mainly comes from ice hydrometeors from convective clouds, which for orographic rain, may not produce much ice aloft and thus, may lead to underestimation of the surface rainfall [73]. A process-based analysis of the IMERG precipitation datasets using cloud top properties sampled from rain gauge and IMERG rainy episodes [61] showed that IMERG frequently misinterprets the warm rains. The authors attributed this to the oversensitivity of IMERG for the low cloud optical thickness and effective radius. The fact that IMERG estimate rainfall from multiple PMW estimates from the GPM constellation could explain the notable differences between the detected rain rates, particularly in the mountainous areas. However, comparatively low elevation areas, notably within the Rift Valley, show low differences instead, probably, due to the low rain rates observed in these areas [49]. The mean differences compared in Figure 12b for the nighttime show similar spatial variability to the daytime, although the differences are comparatively lower than during the daytime.

Figure 12c shows comparable probability densities of the detected rain rates by the gauge, model and IMERG for rain rates below 20 mm per 30 min interval. By contrast, the densities for the above 20 mm rain rates compared in Figure 12d shows that IMERG’s maximum detected rain rate was below 40 mm. On the other hand, the model detections were mainly comparable to the ground truth and above 50 mm per 30 min, suggesting that IMERG has a low tendency to detect very high rain rates. The model’s comparable detected rain rates with the ground truth is because it was calibrated using a dense rain gauge network. IMERG’s low tendency to detecting very high rain rates may be due to the spatial and temporal averaging technique used to merge and intercailibrate rainfall estimates from several PMW sources.

Based on the results, it can be stated that the developed rain area detection and correction technique using multispectral data from the MSG SEVIRI was successful in detecting rain areas at 2 scales: (1) at point and pixel scale when the detected rain areas were compared with gauge station data, and (2) at a large scale when compared to the spatial dynamics of rain areas detected by IMERG. Furthermore, retrieval and comparison of the model, IMERG, and gauge rain rates reveal the model’s capability to detect rain rates comparable to rain gauge data and with better tendency for detecting higher intensities than IMERG.

## 4. Discussion

### 4.1. The Day and Nighttime Rain Detection Technique

The detection of rain areas using multispectral satellite data from MSG SEVIRI was demonstrated for the daytime and nighttime over topographically complex terrain in south-western Kenya. The technique relies on developing and calibrating multiple parametric rain detection models using rainfall data from a dense network of rain gauges to determine the best model parameters and parametric values for successful rain area detection. The models were rooted in the conceptual assumption that clouds characterised by their top properties such as high optical thickness and effective radius (comprising ice and water hydrometeors) have high rainfall probabilities and intensities. Several models in 3 categories: reflectance, IR and combined reflectance-IR models, were developed to answer 2 primary questions: (1) What is the rain detection skill of the proposed spectral models? (2) Which model possesses the best detection skill?

The results, determined using standard categorical statistics and ROC curves, show that over the study area, daytime rain detection based on models using reflectance alone data outperformed those using the IR and combined reflectance-IR models. Combining reflectance and IR data showed medium performance in rain area detection and with the tendency to reduce model FAR and Bias scores. However, the IR-only based models, particularly the model based on a single IR BT, often showed poor performances. The best daytime model was determined from the reflectance models to be VIS0.6–NIR1.6, and the parameter value above which the best detection performance was archived was 0.21. Based on the premise that the VIS0.6 and NIR1.6 reflectance indicates cloud optical thickness and effective radius, respectively and together point towards CWP, the results suggest that the reflectance differences above 0.21 detect clouds with high CWP indicating high rainfall probabilities.

The nighttime models consisted of a single IR BT, BTD and combined IR BT-BTD models developed from IR only spectral data. The results indicate that the BTD model showed the best performance in rain area detection over the study area. In contrast, the combined IR BT-BTD and the single IR BT models showed medium and poor performances, respectively. The best model was determined to be a combination of IR3.9–IR10.8, IR3.9–WV7.3 and IR10.8–WV6.2 BTD. The corresponding parameter values below which the best detection performance was achieved were 8.18 K, 17.03 K and 33.65 K, respectively. The IR3.9–IR10.8 and IR3.9–WV7.3 parameters indicate cloud optical thickness and the IR10.8–WV6.2 BTD indicates the height of the cloud top. Unlike daytime detection, the results suggest that nighttime rain area detection may be best achieved with cloud optical thickness and cloud height information.

Overall, both the day and nighttime models demonstrated high FAR scores that may be due to many factors, such as the non-linear relationship between rainfall and the cloud top properties [66]. The daytime rain area detection performed better than the nighttime, which could be attributed to the relevant information content on CWP and rainfall available in the VIS 0.6 and NIR 1.6 reflectance pair [15]. Furthermore, the bivariate analysis of the raining and non-raining spectral characteristics (Section 3.1.1) and their descriptive statistics (Appendix A) reveal uniqueness in reflectance data that supports its high detection skill. The nighttime model’s comparatively weak performance indicated by its high POFD and low CSI and ETS values was also observed by [15] for a comparatively small area located in the study area. It could be explained in part by the comparable distribution observed for their raining and non-raining spectral characteristics (Section 3.1.1). Nonetheless, the results support the conceptual model that raining clouds characterised by their top properties, such as high optical thickness and effective radius, have high rainfall probabilities.

### 4.2. The Rain Area Correction and Validation Technique

The detected rain areas were corrected for their number (i.e., the number of areas detected as raining) and sizes (i.e., the sizes of the detected rain areas) using rain area-specific parameters and adaptive parametric thresholds. Specifically, two kinds of gradient correction: the cloud object and pixel gradient, were defined for the daytime and nighttime rain area correction. Although their determination differed for the day and nighttime, the implications were the same for both periods. The cloud object gradient reduced the number of areas detected as raining by comparing a rain area-specific cloud gradient with the average gradient and standard deviation from all detected rain areas. On the other hand, the pixel gradient reduced the detected rain areas’ size by comparing the gradient computed for each pixel in a raining area to its median and average median from detected all rain areas.

The developed rain detection parametric model was validated using independent validation sets and comparing the model results to data from the gauge stations and IMERG. The comparison between model rain detection results and the gauge station’s rainfall data showed improvement in daytime detection, indicated by high ACC and low FAR scores compared to its previous calibration results. In contrast, low ETS scores (compared to its previous calibration results) observed for the nighttime detection suggested reduced nighttime detection skill for the validation sets.

The detected rain areas by the parametric model and IMERG were compared to validate the model’s rain area detection skill spatially. Their detected rain rates were compared to the gauge station rainfall data to evaluate the model’s rain rate detection against ground truth and IMERG.

By visual inspection, the agreement in spatial dynamics of detected rain areas by the model and IMERG were generally convincing, particularly for large contiguous raining areas and better during the day than during the nighttime. However, there were some noticeable differences between the detected rain areas. For instance, a slight shift between the detected rain areas by the model and IMERG could be discerned from both the day and nighttime results. Further, IMERG, particularly during the daytime, detected more rain areas of varying sizes, whereas the model’s detection was mainly organised into large contiguous areas. Also, the absolute differences in mean rain rate detected by the model and IMERG at the gauge stations showed somewhat similar spatial dynamics for the day and night. However, the mean differences were comparatively higher for the day than the nighttime.

### 4.3. Uncertainties and Implications of the Rain Area Detection and Correction Technique

The uncertainties in the model results may be related to multiple factors. For instance, the spatial and temporal mismatch between the MSG satellite and gauge observations, although spatiotemporally aggregated following the method by [15,39], impacts the satellite’s data sampled to calibrate and validate the model. Additionally, for multilayered clouds with cloud properties differing between layers [74], the satellite’s information may not agree with the ground observation [75]. This could explain uncertainties found in the bivariate comparison of rain and non-raining spectral characteristics.

Many factors contribute to the differences in rain area and rates detected by the parametric model and IMERG. For instance, the difference in spatial resolution of the MSG and IMERG datasets (approximately 3 × 3 and 10 × 10 km, respectively) suggests that the IMERG data is averaged at a comparatively large spatial resolution than the parametric model; however, in this study, the IMERG pixel was compared to a single gauge. Such a spatial disparity could impact the MAD and could explain for, e.g., the comparatively less number but large contiguous (raining) cloud areas detected by IMERG than the model. Moreover, the developed model was based on reflectance and IR data from a single geostationary satellite, MSG, calibrated using rainfall data from a dense gauge station network. On top of this, the parametric model’s underlying conceptual assumption is the relationship between cloud top information such as high optical thickness and effective radius and rain probability and intensities. IMERG, on the other hand, is a multi-satellite algorithm that combines microwave observations from multiple satellite sensors to estimate half-hourly globally gridded precipitation. Central to IMERG is the morphing technique, which uses motion vectors to fill gaps in passive microwave precipitation estimates using a quasi-Lagrangian interpolation. The latest version of the IMERG rainfall product (V06b, used in this study) uses motion vectors derived from total precipitable water vapour retrieved from numerical models, unlike from geostationary IR BT in the previous versions [30]. This could explain the differences in the number and sizes of the detected rain areas by the model and IMERG.

Furthermore, the shift/dislocation between IMERG relative to the model detected rain areas observed in this study should be due to the procedures undertaken in developing IMERG, such as the morphing passive microwave measurements using the motion vectors [62]. It is worth pointing out that spatial displacement or dislocation errors have not received much attention in satellite rainfall validation. Nevertheless, where the rainfall field is located has significant implications on operational applications’ effectiveness and efficacy, such as flood and flash flood forecasts. In an attempt to address this gap, recent work has suggested some spatial displacement error metrics e.g., [76,77], albeit being applied on reanalysis and rainfall forecast data. However, satellite rainfall evaluations that address this possible spatial displacement are still lacking. The results presented herein implies the possibility of a spatial displacement in satellite rain fields which needs to be investigated, quantified and corrected. Therefore, future studies will investigate and address this potential spatial displacement error in rain areas from satellites.

Quite apart, the rainfall and satellite datasets used in this study represent a significantly larger space and time domain compared to the previous study within the area [15]. Thus, the developed model and parameters may be regionally applicable in the tropical (Eastern) African region to detect and correct rain areas using multispectral geostationary satellite data. Nevertheless, studies such as [7] suggest that the rain area detecting parameters are regional and climate-dependent. Therefore, more research on the technique’s applicability over a comparatively more extensive scale, e.g., continental, must be completed until a final rain area detection scheme will be available. In this regard, the near continental scale coverage of TAHMO’s stations in Africa [27] and the existence of opportunistic rain sensors such as CML [78] are, for e.g., two valuable sources of rainfall information that heightens the potential of developing such a spatiotemporally high detection scheme. Despite the challenges e.g., [79], it has been well established, in the last decade, that accurate near ground rainfall monitoring using CML data is possible and could benefit the sparsely gauged regions or complement conventional monitoring techniques. The study will be extended to cover sub-Saharan Africa and match TAHMO’s spatial coverage in a future step. In addition, cloud top information from Cloud Property Dataset Using SEVIRI, edition 2 (CLAAS-2) [80] will replace the indirect cloud-top property information inferred from the SEVIRI optical, near and thermal IR data in this study. This could potentially reduce uncertainties that were due to analysing ground rainfall with indirect cloud top information.

## 5. Conclusions

A gradient based adaptive technique capable of day and nighttime rain area detection and correction is presented using reflectance and IR data from the MSG SEVIRI satellite observations from south-western Kenya. In this investigation, we first developed, calibrated and validated multiple parametric rain detection models using rainfall data from a dense gauge station network to determine the best model parameters for the day and nighttime. We then developed a new technique to correct the detected rain areas—the method uses rain area-specific parameters to reduce the number and sizes of the detected rain areas.

Compared to the GPM IMERG and gauge station data, the developed model shows convincing agreement in both the detected rain area and rain rates, suggesting that the new technique could provide valuable insights to satellite rainfall retrievals to benefit many operational applications.

The technique’s limitation is related to the fact that it calibrates the satellite data using gauge rainfall data which may not be available everywhere. Additionally, the rain detecting parameters identified from the satellite data may be regionally and climate-dependent—implying the technique should be calibrated per study region to obtain suitable parameter values for a successful rain area detection. In that sense, the study by [15] is for, e.g., a proof of concept that near ground rainfall from microwave attenuations on CML can be used in place of the gauge data to overcome these limitations.

## Figures and Tables

**Table 1:** Spectral characteristics and cloud top parameters used for rain detection.

Spectral Characteristics	Inferred Cloud Top Parameter	Application
VIS0.6 and NIR1.6	Optical thickness and effective radius	Daytime
IR10.8 K	Cloud top temperature	Day and nighttime
IR10.8–WV6.2 K	Height	Day and nighttime
IR10.8–IR12.0 K	Phase (water)/optical thickness	Day and nighttime
IR8.7–IR10.8 K	Phase (ice)	Day and nighttime
IR3.9–IR10.8 and IR3.9–WV7.3 K	Optical thickness and effective radius	Nighttime
WV6.2–WV7.3 K	Level (high or low)	Day and night time

**Table 2:** Summary of non-zero rainfall from calibration and validation sets for the daytime and nighttimes from the long rain period of 2018–2020.

	Calibration		Validation	
	Day	Night	Day	Night
Mean mmh^−1^	5.59	4.39	5.26	4.00
Maximum mmh^−1^	157.01	137.26	81.87	97.89
Standard deviation	8.97	6.32	7.42	5.47
Fraction %	13.47	16.35	13.21	14.93
N	5111	9457	1544	2957
n days	212	209	104	101

N is the total number of 30 min aggregated intervals assembled from an n (313 days) number of daytime and nighttime rainy days during the evaluation period. The Fraction (%) represents the percentage of the non-zero rain rates in entire datasets (i.e., including zero and non-zero rain rates).

**Table 3:** The BTD combinations investigated for rain area detection.

BTD Combinations	
BTD1	(IR10.8–IR12.0) and (IR8.7–IR10.8)
BTD2	(IR10.8–IR12.0) and (IR8.7–IR10.8) and (WV6.2–WV7.3)
BTD3	(IR10.8–IR12.0) and (IR8.7–IR10.8) and (IR10.8–WV6.2)
BTD4	(IR3.9–IR10.8) and (IR3.9–WV7.3) and (IR10.8–WV6.2)
BTD5	(IR10.8–IR12.0) and (IR3.9–WV7.3) and (IR10.8–WV6.2)
BTD6	(IR10.8–IR12.0) and (WV6.2–WV7.3) and (IR8.7–IR10.8)

**Table 4:** The categories of rain area detection and their parametric models for the day and night times.

Period	Reflectance-Only		IR-Only	Combined Reflectance-IR
Daytime	Ref 1	VIS0.6 and NIR1.6	IR10.8 and BTD1	Ref 1 and BTD3
	Ref 2	VIS0.6 ÷ NIR1.6	IR10.8 and BTD2	Ref 2 and BTD2
	Ref 3	VIS0.6 − NIR1.6	IR10.8 and BTD3	Ref 3 and BTD1
			IR 10.8	
Nighttime			BTD4	
			IR10.8 and BTD5	
			IR10.8 and BTD6	
			IR10.8	

Note: Ref is a reflectance model.

**Table 5:** Contingency table for evaluating the raining and non-raining decision.

2 × 2 Contingency Table		Gauge Observation	
		Raining	Non-Raining
**Model observation**	Raining	h	f
	Non-raining	m	z

**Table 6:** Summary of the categorical statistics.

Statistic	Equation	Range	Optimal Value
POD	$\frac{h}{h+m}$	[0,1]	1
FAR	$\frac{f}{h+f}$	[0,1]	0
POFD	$\frac{f}{z+f}$	[0,1]	0
Bias	$\frac{h+f}{h+m}$	[0,∞]	1
ACC	$\frac{h+z}{ɳ}$	[0,1]	1
CSI	$\frac{h}{h+m+f}$	[0,1]	1
ETS	$\frac{\left(h-h_{erandom}\right)}{h+m+f-h_{erandom}}$	[−1/3,1]	1

where ɳ=h+m+f+z and $h_{erandom}=\frac{\left(h+m\right)\times \left(h+f\right)}{ɳ}$.

**Table 7:** Best rain detection model and the parameter values during the day and nighttime.

Application	Rain Detection Model	Parameter	Parameter Value
Daytime	Ref 3	VIS0.6–NIR1.6	0.21
Nighttime	BTD4	IR3.9–IR10.8	8.18 K
		IR3.9–WV7.3	17.03 K
		IR10.8–WV6.2	33.65 K

**Table 8:** Descriptive statistics of spatial properties of the rain areas detected per 30 min time step.

Descriptive Statistics	Number Of Contiguous Cloudy Areas			Area (km^2^)		
	Uncorrected Model	Corrected Model	GPM IMERG	Uncorrected Model	Corrected Model	GPM IMERG
Average	186	85	19	2522	814	2275
50%	120	63	16	1732	621	1545
75%	195	103	26	3230	988	2863
Stand. dev.	191	86	15	2453	795	2547

Stand. dev. is standard deviation, 50% and 75% are the percentile values.

## Appendix A

The tables in this section are the descriptive statistics of the spectral characteristics during the daytime. The notations: Stan. Dev, 25%, 50%, 75% are standard deviation, percentile values respectively.

**Table A1:** Descriptive statistics of the spectral characteristics of raining cloud top properties.

Descriptive Statistics	VIS0.6	NIR1.6	VIS0.6/NIR1.6	VIS0.6–NIR1.6	IR 10.8	IR10.8–WV6.2	IR8.7–IR10.8	IR10.8–IR12.0	WV6.2–WV7.3
Minimum	0.06	0.01	0.46	−0.11	213.02	0.38	−3.61	−0.95	−27.82
Maximum	1.00	0.81	17.47	0.90	288.02	50.01	5.49	6.56	0.87
Mean	0.63	0.27	2.51	0.35	253.03	18.89	0.16	1.01	−10.58
Mode	0.70	0.20	1.60	0.40	262.70	23.80	−0.80	0.50	−14.80
Median	0.65	0.26	2.40	0.36	256.24	19.09	0.05	0.81	−11.42
Stan. Dev	0.21	0.11	1.02	0.18	16.51	10.30	1.11	0.89	4.93
25%	0.50	0.20	1.78	0.22	241.42	10.86	−0.68	0.43	−14.56
50%	0.65	0.26	2.40	0.36	256.24	19.09	0.05	0.81	−11.42
75%	0.77	0.34	3.10	0.49	264.96	25.74	0.83	1.38	−6.78

**Table A2:** Descriptive statistics of the spectral characteristics of non-raining cloud top properties.

Descriptive Statistics	VIS0.6	NIR1.6	VIS0.6/NIR1.6	VIS0.6–NIR1.6	IR 10.8	IR10.8–WV6.2	IR8.7–IR10.8	IR10.8–IR12.0	WV6.2–WV7.3
Minimum	0.03	0.01	0.24	−0.31	212.49	0.15	−4.04	−9.31	−23.84
Maximum	1.00	0.89	10.67	0.78	294.91	57.02	8.27	15.10	2.49
Mean	0.43	0.32	1.39	0.11	266.62	29.86	−0.10	1.96	−14.34
Mode	0.40	0.30	1.00	0.00	268.90	30.10	−1.00	0.70	−16.60
Median	0.42	0.32	1.22	0.07	268.93	30.42	−0.40	1.70	−15.24
Stan. Dev	0.20	0.12	0.69	0.16	14.46	10.75	1.28	1.34	3.97
25%	0.27	0.24	0.95	−0.02	260.23	22.98	−1.02	0.92	−17.03
50%	0.42	0.32	1.22	0.07	268.93	30.42	−0.40	1.70	−15.24
75%	0.58	0.41	1.61	0.20	277.23	38.10	0.54	2.75	−12.67

## Appendix B

Table A3 and Table A4 are analogous to those in Appendix A but during the nighttime. The notations in Appendix B have the same meaning in Appendix A.

**Table A3:** Descriptive statistics of the spectral characteristics of raining cloud top properties.

Descriptive Statistics	IR3.9–IR10.8	IR3.9–WV7.3	IR 10.8	IR10.8–WV6.2	IR8.7–IR10.8	IR10.8–IR12.0	WV6.2–WV7.3
Minimum	−7.57	−1.26	220.10	2.19	−1.89	−0.72	−22.84
Maximum	21.13	33.06	285.66	48.16	7.15	8.47	−0.70
Mean	1.91	10.54	254.93	20.41	0.18	1.21	−11.78
Mode	−1.50	8.50	258.40	17.10	0.20	0.70	−14.70
Median	1.16	9.61	256.04	20.30	0.04	0.99	−12.12
Stan. Dev	3.72	4.37	12.52	7.97	1.07	0.85	3.72
25%	−0.96	7.45	245.62	14.43	−0.67	0.63	−14.63
50%	1.16	9.61	256.04	20.30	0.04	0.99	−12.12
75%	4.17	12.71	264.73	26.00	0.82	1.56	−9.09

**Table A4:** Descriptive statistics of the spectral characteristics of non-raining cloud top properties.

Descriptive Statistics	IR3.9–IR10.8	IR3.9–WV7.3	IR 10.8	IR10.8–WV6.2	IR8.7–IR10.8	IR10.8–IR12.0	WV6.2–WV7.3
Minimum	−9.38	1.22	220.10	2.12	−2.20	−0.45	−23.89
Maximum	28.00	37.97	289.38	51.20	6.29	39.32	4.57
Mean	2.68	15.35	261.92	26.69	0.09	1.80	−14.01
Mode	−1.90	11.80	267.10	28.50	−1.00	0.80	−15.40
Median	1.81	14.37	264.02	27.22	−0.13	1.58	−14.65
Stan. Dev	4.68	6.21	12.08	8.50	1.22	1.13	3.49
25%	−0.97	10.49	254.78	20.96	−0.90	0.94	−16.54
50%	1.81	14.37	264.02	27.22	−0.13	1.58	−14.65
75%	5.41	19.48	270.42	32.78	0.86	2.44	−11.98
